# Evaluating stillborn and litter size as indicators of PRRSV detection in live piglets and the use of stillborn tongue fluids as risk-based samples for PRRSV monitoring

**DOI:** 10.3389/fvets.2025.1600064

**Published:** 2025-05-27

**Authors:** Isadora F. Machado, Peng Li, Jinnan Xiao, Thomas Petznick, Ana Paula P. Silva, Onyekachukwu H. Osemeke, Lucina Galina Pantoja, Phillip Gauger, Giovani Trevisan, Gustavo S. Silva, Daniel C. L. Linhares

**Affiliations:** ^1^Department of Veterinary Diagnostic and Production Animal Medicine, College of Veterinary Medicine, Iowa State University, Ames, IA, United States; ^2^Genus plc PIC, Hendersonville, TN, United States

**Keywords:** tongue fluids, risk-based, PRRSV, monitoring, targeted sampling, swine

## Abstract

**Introduction:**

A risk-based approach to animal selection for sampling enhances pathogen detection by increasing the probability of selecting an animal harboring the pathogen while requiring a smaller sample size. Postmortem tongue fluids (TF) have emerged as a promising risk-based approach, with a PRRSV RNA positivity rate similar to serum, processing fluids, and family oral fluids. Thus, this study assessed the effect of stillborn presence, litter size, and PRRSV RNA detection by RT-qPCR in stillborn TF on the probability of having viremic piglets within the litter.

**Methods:**

Samples from 130 litters were collected within 12 hours after farrowing from two breeding herds. TF and intracardiac blood were collected from stillborns, and tail blood swabs were collected from liveborn littermates within the selected litters. Samples were individually tested for PRRSV RNA detection by RT-qPCR. Litters with ≤ 11 liveborn piglets were defined as small. Generalized linear regression models were used to evaluate the litter size, presence of stillborns, and stillborn PRRSV results on the probability that a litter or at least one liveborn littermate would test PRRSV-positive.

**Results:**

The live piglets’ mean positivity within the litter was 5.0%, while the total born was 4.6%. Litters with at least one stillborn had 12.5 times higher odds of having a PRRSV-positive result, and 4.8 times higher odds of having at least one viremic liveborn piglet. In small litters, the odds of having a PRRSV-positive result increased 12.2 times, whereas the odds of having a viremic liveborn littermate increased 10.8 times. When the stillborn TF was positive, the odds of having a viremic liveborn littermate increased 17.6 times.

**Discussion:**

In conclusion, stillborn TFs were a reliable indicator of PRRSV status among litters. Liveborn piglets from litters with PRRSV-positive stillborn TF or small litters had greater odds of testing PRRSV-positive. Therefore, stillborn TF collection and targeting small litters improve PRRSV detection and support farrowing room biocontainment strategies.

## 1 Introduction

Surveillance through periodic collection of porcine biological samples is essential for objectively classifying breeding herds undergoing porcine reproductive and respiratory syndrome virus (PRRSV) control and elimination (1). Specifically, as the virus prevalence within a herd declines, identifying PRRSV circulation by reverse transcription polymerase chain reaction (RT-qPCR) testing requires a larger sample size and more sensitive sample types to ensure timely detection (2), which highly encouraged researchers to develop population-based samplings for a wider population screening (3–6). However, PRRSV-positive pigs are often clustered in barns, with non-homogeneous distribution among the population (7, 8). To overcome this challenge, targeted sampling, also known as risk-based sampling, can be adopted (9).

Risk-based sampling involves stratifying the source population based on characteristics associated with the probability of pathogen occurrence (10, 11). Thus, the advantage of risk-based sampling lies in its efficiency (i.e., it maximizes the probability of detection), particularly when the disease occurs at low prevalence rates (9). For instance, in the context of PRRSV, younger parities are more susceptible to the virus than older parities (12), and PRRSV can cause embryonic death in early gestation, clinical manifestation in late gestation, and is characterized by increased abortions and stillborn piglets, fetal death, and early farrowing (13–15).

Population-based samples combined with a risk-based approach have been reported as an effective method to enhance on-farm PRRSV detection with a lower sample size than traditional recommendations. For instance, collecting family oral fluids (FOF), a sample originating from suckling litters before weaning, from young parity litters (≤ 2 parity) or small litter sizes (≤ 11 piglets) can increase the odds of detecting PRRSV by 3.4 and 9.9 times, respectively (7, 16). Similarly, Vilalta et al. (17) reported that collecting processing fluids (PF), a fluid recovered from tissues collected at castration from piglets, from young parity female litters should be prioritized for PRRSV RNA detection by RT-qPCR. Following this concept, tongue fluid (TF) appears to align with the risk-based category, as it targets dead animals (6). Moreover, it has been described as an alternative population-based sample with similar positivity to FOF, PF, and serum samples (18). Also, Dürlinger et al. (19) reported in a longitudinal field study that TF had the highest viral load when examined at a litter level compared to serum, PF, and oral fluids.

Since PRRSV can be transmitted from infected sows to their fetuses during late gestation, vertical transmission represents an important source of PRRSV spread within swine populations (20, 21). However, a significant gap in the current monitoring programs is the lack of a convenient, population-based approach to detect PRRSV circulation within a gestating population. An alternative approach that might be used to indirectly measure PRRSV circulation in the gestating population is by collecting samples from the offspring that were possibly vertically infected. TF collected from stillborn piglets were reported as a well-suited sample for detecting vertical transmission within the herds, with a strong positive correlation and high level of agreement in PRRSV RNA load in TF and serum from stillborn piglets (19, 22). Moreover, collecting TF from stillborn pigs was demonstrated to have a higher PCR positivity and lower cycle threshold (Ct) value than serum from liveborn littermate pigs and PF from the same litter (23). However, less is known about how PRRSV-RNA positive results in stillborn piglets reflect the PRRSV status of their liveborn littermates, suggesting an important information gap, as it potentially contributes to the further virus spreading across farrowing room populations.

Therefore, based on PRRSV dynamics, such as increased stillborn rates and decreased liveborn piglets per sow, this study evaluated PRRSV monitoring programs’ effectiveness in using TF from stillborn piglets and how results reflected liveborn littermates’ PRRSV status in commercial breeding herds. Secondly, this study assessed the probability of a PRRSV RNA-positive result in litters with at least one stillborn compared to those without and the association of litter size on PRRSV-RNA detection within the litter.

## 2 Materials and methods

### 2.1 Study design

A cross-sectional field study was conducted to estimate the association of the presence of stillborn piglets, litter size, and stillborn TF results on the probability of having viremic piglets in the litter. From two PRRSV-unstable breeding herds (2), samples were collected from liveborn and stillborn piglets from 130 litters within 12 h after farrowing to assess PRRSV status at birth. TF and intracardiac blood (IB) were collected from the stillborn piglets, and tail blood swabs (BS) were collected from the liveborn littermates. Samples were individually tested for PRRSV RNA by RT-qPCR by the investigator.

Generalized linear regression models were used to evaluate the following: whether the presence of stillborn piglets had an effect on detecting PRRSV in the litter and liveborn piglets; if PRRSV RT-qPCR results from stillborn TF and IB were indicators of PRRSV status in liveborn piglets; and whether litter size impacted the probability that at least one live littermate tested PRRSV-RNA positive. This study was approved by the Institutional Animal Care and Use Committee (IACUC) of Iowa State University, IA, USA, under protocol IACUC-22-101 “Field surveillance for swine pathogens” and IBC-24-096 “Field surveillance of swine pathogens using post-mortem tissues”.

### 2.2 Inclusion criteria for breeding herd selection

To obtain a satisfactory proportion of PRRSV RNA-positive samples from piglets, two PRRSV-positive unstable breeding herds (Herds A and B) in the Midwestern USA undergoing PRRSV elimination were selected based on weekly PRRSV RNA detection in PF from the suckling pig population. Both herds broke with PRRSV-2 RFLP 1–8-4 Lineage 1H (24) and immediately underwent live virus inoculation (LVI) protocols after PRRSV-2 wild-type detection. Herd A was a 2,500-sow farm with a four-week batch farrowing system that received LVI 120 days before the first sampling day; Herd B was a 7,000-sow farm with a weekly batch farrowing system that received LVI 107 days before the first sampling day.

### 2.3 Sample collection and processing

Over a consecutive period of 5 days in Herd A and 9 days in Herd B, within 12 h of farrowing, a total of 130 litters from both herds were sampled (26 litters from Herd A and 104 from Herd B): 66 litters without stillborn piglets and 64 with stillborn piglets, totaling 1,723 liveborn and 105 stillborn sampled piglets. Both herds had been actively assisted farrowing by farm personnel. Throughout the study, the same trained investigator performed the stillborn piglet sampling, while two performed the liveborn piglet sampling. All liveborn and stillborn piglets within each litter were sampled. Stillborn piglets were defined as those found dead at birth, often presenting a brown-greenish colour, discoloured skin, or retention within the placenta, with intact thimbles covering their feet. Mummified piglets, defined as fetuses that died in utero and exhibited signs of decomposition such as dehydration and sunken eyes, were not sampled.

Tongue tips were collected from all stillborn piglets within the sampled litters. Briefly, three centimetres of each stillborn tongue tip was collected using sterile disposable scalpel blades (Size 20, Securos Surgical, Fiskdale, MA, USA), individually placed in 50 mL centrifuge tubes (Thermo Fisher Scientific, Pittsburgh, PA, USA) containing 1 mL of phosphate buffered saline (PBS) (PBS 1x, RPI Research Products International, Mt. Prospect, IL, USA), followed by a freezing process under-20°C for 24h. Gloves and scalpel blades were discarded after each stillborn sampling. The samples were thawed at 4°C for 6 h, the fluid was extracted, placed into 5 mL tubes (Thermo Fisher Scientific, Pittsburgh, PA, USA), and frozen at-20°C until laboratory diagnostic testing.

Intracardiac blood (IB) from stillborn piglets were collected directly from the piglet’s heart using a single-use 5 mL syringe (Monoject^TM^, Cardinal Health, Waukegan, IL, USA) and a single-use 20G x 1” needle (Monoject^TM^, Covidien, Mansfield, MA, USA) and kept at 4°C until laboratory shipment. In the laboratory, IB samples were centrifuged and sera were transferred into 5 mL tubes (Thermo Fisher Scientific, Pittsburgh, PA, USA) before diagnostic testing.

From the liveborn piglets, tail blood swabs (BS) were collected using cotton-tipped swabs (Puritan Medical Products Company, LLC, Guilford, ME, USA) and disposable scalpel blades (Size 20, Securos Surgical, Fiskdale, MA, USA) from the piglets’ tails after the tail docking process, and swabs were then placed into 5 mL tubes (Thermo Fisher Scientific, Pittsburgh, PA, USA) containing 1 mL of PBS (PBS 1x, RPI Research Products International, Mt. Prospect, IL, USA). Scalpel blades were discarded after each piglet sampling. BS samples were frozen at-20°C before diagnostic testing.

#### 2.3.1 PRRSV RNA extraction and PRRSV RT-qPCR

All samples were individually tested using RT-qPCR in the research facilities of a National Animal Health Laboratory Network-accredited veterinary diagnostic laboratory at Iowa State University. Briefly, nucleic acids were extracted from the samples using the RealPCR*DNA/RNA Magnetic Bead Kit (IDEXX Laboratories, Inc., Westbrook, ME, USA), following the manufacturer’s instructions and automated extraction equipment (Kingfisher Flex System Magnetic Beads Processor, Thermo-Fisher Scientific, Waltham, MA, USA) (25). A positive amplification control (IDEXX Laboratories, Inc., Westbrook, ME, USA), a negative amplification control (nuclease-free water), a positive PRRSV-2 extraction control sample, and a PRRSV-2 negative extraction control sample were included for each RT-qPCR plate (25). According to the manufacturer’s recommendations, samples with Ct values < 40 were considered PRRSV-positive.

### 2.4 Statistical analysis

Data analyses were conducted in R program software (version 4.1.2). The Cohen Kappa agreement test was performed between TF and IB PRRSV RT-qPCR testing results at an animal level. Multivariate generalized linear regression models were used to estimate the effects of stillborn presence, stillborn PRRSV-RNA result (IB and TF), and litter size on the probability that a litter or at least one liveborn littermate would test PRRSV-positive (five analyses in total). For these analyses, the variables were classified as categorical: the presence of at least one stillborn piglet within the litter (presence, absence), at least one PRRSV-positive stillborn piglet (yes, no), at least one TF PRRSV-positive within the litter (yes, no), at least one IB PRRSV-positive within the litter (yes, no), and litter size (small, large). Litters with ≤ 11 liveborn piglets were defined as small litters based on the first quantile (the lowest 25%) for liveborn piglets registered in both herds during the collection period, whereas litters with ≥ 12 were defined as large. The “Herd” identifier was initially tested as a random effect in a generalized linear mixed regression. The model variance–covariance resulted in 0, and ‘Herd’ was excluded since there was no evidence of hierarchical structure. Model assumptions were assessed using the Wald test, deviance analysis, and Goodness-of-Fit test. Non-parametric analysis was conducted using Kruskal-Wallis and Dunn tests to assess the Ct values differences between the sample types. A *p*-value < 0.05 was used to determine the statistical significance of all analyses.

## 3 Results

A total of 1,723 liveborn piglets and 105 stillborn piglets were individually sampled from 130 litters from two herds: 26 litters from Herd A and 104 from Herd B (Table 1). Results from Herds A and B were simultaneously analyzed. Regarding the litter’s parity: in Herd A, 14 were parity 1 sows, seven were parity 2, four were parity 3, and one parity 4; in Herd B, all 104 litters came from parity 1 sows.

**Table 1:** Descriptive information for herds, litters, and piglets sampled for tail blood swabs, intracardiac blood, and tongue fluids.

Herd	Sows (*n*)	Sampled litters			Tail Blood Swab^1^ (*n*)			Intracardiac Blood^2^ (*n*)			Tongue Fluids^3^ (*n*)		
		Without stillborn	With stillborn	Total	Negative	Positive	Total	Negative	Positive	Total	Negative	Positive	Total
A	2,500	8	18	26	372	7	389	34	2	36	31	5	36
B	4,500	58	46	104	1,282	52	1,334	54	15	69	46	23	69
AB	7,000	66	64	130	1,654	59	1,723	88	17	105	77	28	105

^1^Tail Blood Swabs (BS) were collected on liveborn piglets; 20 of 130 litters had at least one positive test result for PRRSV RNA.

^2^Intracardiac Blood (IB) samples were collected on stillborn piglets; 13 of 130 litters had at least one positive test result for PRRSV RNA.

^3^Tongue Fluid (TF) samples were collected on stillborn piglets; 22 of 130 litters had at least one positive test result for PRRSV RNA.

Considering all sample types (TF, IB, and BS), the percentage of PRRSV-positive litters based on PRRSV RNA testing was 22.3% (29 of 130 litters), of which 15.4% (20 of 130 litters) were BS PRRSV RNA-positive, 10% (13 of 130 litters) were IB PRRSV RNA-positive, and 16.9% (22 of 130 litters) were TF PRRSV RNA-positive. At an animal-level, considering both herds, 3.4% (59 of 1,723 liveborn piglets) were BS PRRSV-RNA positive, 16.2% (17 of 105 stillborn piglets) were IB PRRSV-RNA positive, and 26.7% (28 of 105 stillborn piglets) were TF PRRSV-RNA positive (Table 1). The mean positivity of liveborn piglets within the litter was 5%, varying from 0 to 85.7%, while the total born (stillborn and liveborn piglets combined) was 4.6% (0 to 76.4%).

**Figure 1:**
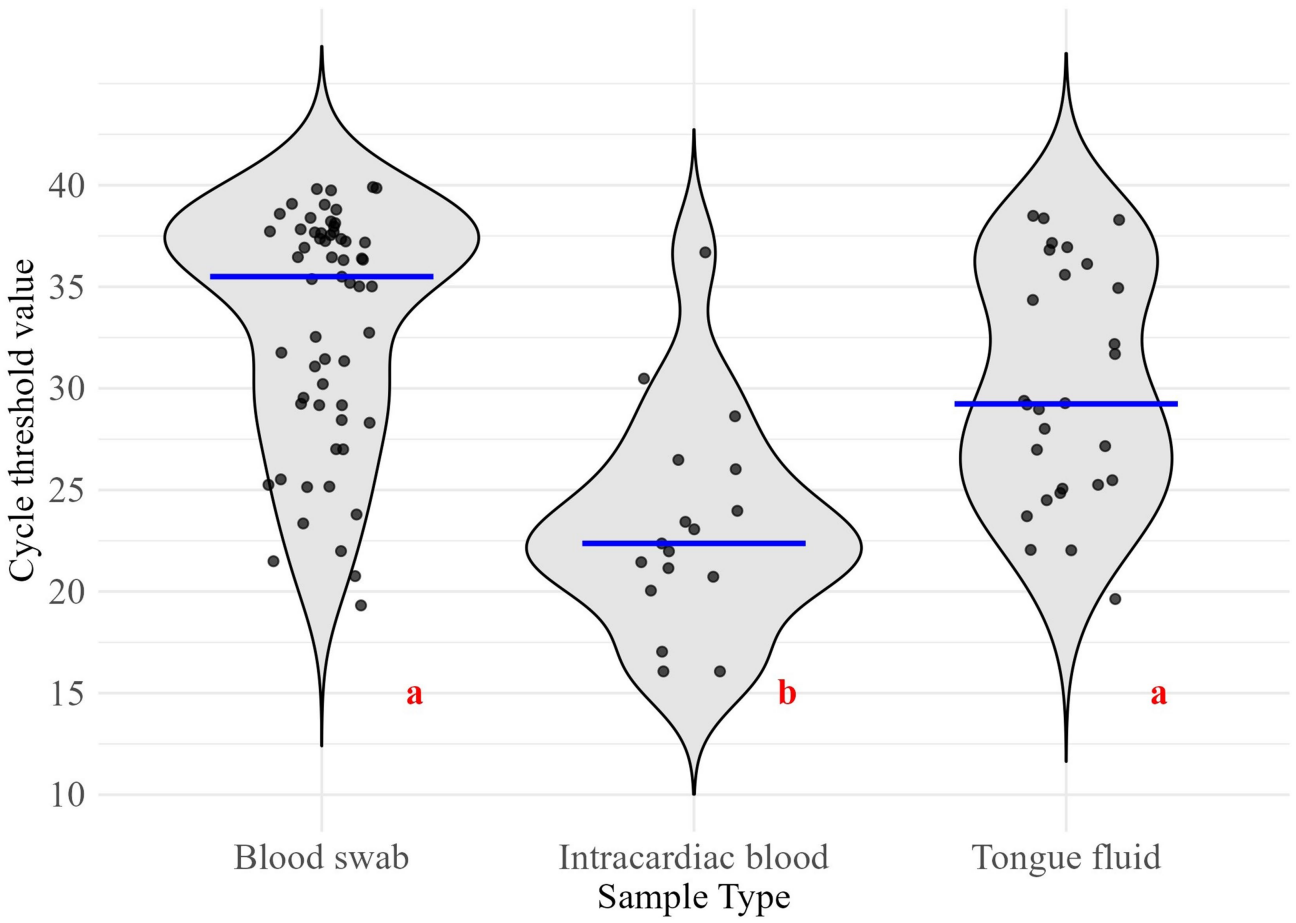
Distribution of cycle threshold values for the collected sample types. Different red letters indicate significant differences (*α* = 0.05). Blue lines represent group medians. Sample sizes for PRRSV-positive results: Blood swab (*n* = 59), Intracardiac blood (*n* = 17), Tongue fluid (*n* = 28).

Regarding sample types’ Ct values, BS had a median of 35.5 (interquartile range [IQR]: 29.2–37.7), IB a median of 22.4 (IQR: 20.7–26.0), and TF a median of 29.2 (IQR: 25.2–35.7) (Figure 1). IB exhibited the lowest median Ct value, which was significantly different from the median Ct values of the compared sample types (*p*-value < 0.05).

At the animal level, TF and IB had a substantial agreement (Kappa = 0.694, *p*-value < 0.001). Results of the five multivariate logistic regression models are available in Table 2, and their predicted probabilities of PRRSV-RNA detection in litters or liveborn piglets by the assessed predictor variables (litter size, presence of stillborn piglet, and stillborn piglet PRRSV result [TF, IB, or both]) are available in Table 3.

**Table 2:** Multivariable logistic regression analyses of predictors for PRRSV-RNA detection in litters or liveborn littermates.

Model	Outcome	Predictor	Odds ratio	95% CI^1^	*p*-value
1	Positive litter^2^	Reference: large litter or litter without SB^4^	1	–	
		Small litter	12.22	4.00, 46.49	< 0.001
		SB^4^ present	12.53	3.93, 51.97	< 0.001
2	Positive liveborn littermate^3^	Reference: large litter or litter without SB^4^	1	–	
		Small litter	10.78	3.60, 36.55	< 0.001
		SB^4^ present	4.77	1.54, 17.14	0.005
3	Positive liveborn littermate^3^	Reference: large litter or litter without SB^4^	1	–	
		Small litter	7.00	2.02, 28.84	0.002
		SB^4^ TF^5^ or IB^6^ negative	0.42	0.02, 2.94	< 0.001
		SB^4^ TF^5^ or IB^6^ positive	17.61	4.89, 78.67	
4	Positive liveborn littermate^3^	Reference: large litter or litter without SB^4^	1	–	
		Small litter	7.00	2.02, 28.84	0.002
		SB^5^ TF^6^ negative	0.42	0.02, 2.94	< 0.001
		SB^5^ TF^6^ positive	17.61	4.89, 78.67	
5	Positive liveborn littermate^3^	Reference: large litter or litter without SB^4^	1	–	
		Small litter	7.91	2.38, 29.89	< 0.001
		SB^5^ IB^7^ negative	1.85	0.46, 7.60	< 0.001
		SB^5^ IB^7^ positive	26.34	5.62, 157.67	

^1^95% CI: 95% confidence interval. ^2^Positive litter: at least one PRRSV positive BS, IB, or/and TF. ^3^Positive liveborn littermate: at least one PRRSV-positive liveborn littermate. ^4^SB: stillborn. ^5^TF: tongue fluids. ^6^IB: intracardiac blood.

**Table 3:** Predicted probabilities of PRRSV-RNA detection in litters or liveborn piglets by the assessed predictors.

Model	Outcome	Predictor	Probability of PRRSV RNA^1^ detection	95% CI^2^	*p*-value*
1	Positive litter^3^	SB^5^ absent	8.02%	3.2, 35.6%	< 0.001
		SB^5^ present	52.21%	10.7, 43.6%	
		Large litter	8.11%	3.8, 16.3%	< 0.001
		Small litter	51.90%	32.4, 70.8%	
2	Positive liveborn littermate^4^	SB^5^ absent	8.27%	3.4, 18.7%	0.010
		SB^5^ present	30.11%	17.9, 46.0%	
		Large litter	5.66%	2.5, 12.5%	< 0.001
		Small litter	39.29%	23.7, 57.5%	
3	Positive liveborn littermate^4^	Large litter	6.84%	2.6, 16.7%	0.003
		Small litter	33.98%	15.2, 59.6%	
		SB^5^ TF^6^ or IB^7^ negative	4.06%	0.6, 24.1%	0.002
		SB^5^ TF^6^ or IB^7^ positive	63.64%	40.0, 82.1%	
4	Positive liveborn littermate^4^	Large litter	3.62%	1.06, 11.6%	0.003
		Small litter	20.82%	9.1, 40.8%	
		TF^6^ SB^5^ negative	4.06%	0.6, 24.1%	0.002
		TF^6^ SB^5^ positive	63.64%	40.0, 82.1%	
5	Positive liveborn littermate^4^	Large litter	3.33%	0.9, 10.7%	0.001
		Small litter	21.43%	9.5, 41.3%	
		IB^7^ SB^5^ negative	15.26%	6.3, 32.5%	0.004
		IB^7^ SB^5^ positive	71.87%	40.5, 90.6%	

^1^PRRSV RNA: porcine reproductive and respiratory syndrome virus RNA detection by reverse transcriptase polymerase chain reaction. ^2^95% CI: 95% confidence interval. ^3^Positive litter: at least one PRRSV positive BS, IB, or/and TF. ^4^Positive liveborn littermate: at least one PRRSV-positive liveborn littermate. ^5^SB: stillborn. ^6^TF: tongue fluids. ^7^IB: intracardiac blood. *Results are averaged over the levels of included covariates.

In model 1, small litters had 12.2 times higher odds (95% CI: 4.00 – 46.49, *p*-value < 0.001) of having a PRRSV-positive result compared to large litters, holding the stillborn variable constant, with a 51.9% probability of detecting PRRSV-positive piglets in small litters compared to 8.1% in large litters. Litters with at least one stillborn piglet had 12.5 times higher odds (95% CI: 3.93, 51.97, *p*-value < 0.001) of having a PRRSV-positive piglet, holding litter size constant, with a 52.2% probability of detecting a PRRSV-positive piglet when at least one stillborn was present, compared to 8.0% when no stillborns were present. No significant interaction between litter size and stillborn presence was found (*p*-value = 0.076). In model 2, small litters had 10.8 times higher odds (95% CI: 3.60 – 36.55, *p*-value < 0.001) of having a viremic liveborn littermate compared to large litters, holding the stillborn variable constant. Litters with at least one stillborn piglet had 4.8 times higher odds (95% CI: 1.54, 17.14, *p*-value = 0.005) of having a viremic liveborn littermate, compared to litters without stillborn piglets, holding litter size constant. Further probabilities to detect PRRSV-positive liveborn in the litter are described in Table 3.

Stillborn PRRSV RNA results were included as predictor variables in models 3, 4, and 5, along with litter size. Model 3 included both TF and IB results, model 4 included the TF only, and model 5 IB only. Litters with PRRSV-positive stillborn piglets – detected either by combined TF or IB, or TF only – had 17.6 times higher odds (95% CI: 4.89, 78.67, *p*-value < 0.001) of having a viremic liveborn littermate compared to those without stillborn piglets, holding litter size constant. Small litters had 7.0 times higher odds (95% CI: 2.02, 28.84, *p*-value = 0.002) of having a viremic liveborn littermate compared to large litters, holding the stillborn PRRSV result constant. Further probabilities to detect PRRSV-positive liveborn in the litter are described in Table 3.

## 4 Discussion

The US swine industry continuously updates its PRRSV surveillance programs, incorporating standardized systems for PRRSV classification and sample type recommendations (1, 2). Surveillance and classification systems are highly reliant on diagnostic testing and play an important role in facilitating communication between veterinarians and swine producers. They also support the implementation of regional and national efforts toward PRRSV control and elimination. Over the years, multiple population-based sample types, such as PF, oral fluids, FOF, and TF, were developed to improve the monitoring programs and to overcome labor and cost issues (3–6). As a result, the AASV PRRSV classification committee incorporated both individual and population-based samples in its latest recommendations for a broader population coverage and provided additional evidence to increase confidence in detecting PRRSV-positive pigs (2). Similarly, risk-based approaches have been reported to be associated with an increased probability of detecting viral infections (16, 17). Thus, using a risk-based approach, the current study provided important insights into detecting PRRSV RNA across multiple sample types from liveborn and stillborn piglets.

PRRSV is known to cause negative reproductive impacts. When it infects the gestating population, it can cause an increase in abortions, fetal death, and stillborn piglet rates (26, 27). When horizontally transmitted, PRRSV can cause viremia as early as 12 h post-exposure (28–30). In this study, litters with at least one stillborn piglet had 12.5 times higher odds of testing PRRSV RNA-positive (based on IB, TF, or BS) within 12 h of farrowing than those without stillborns. Additionally, the odds of having a viremic liveborn littermate were 4.8 times higher in such litters, and when the stillborn tested TF PRRSV RNA-positive, the odds of finding a viremic liveborn littermate increased dramatically by 17.6 times. Thus, stillborn piglet occurrence can be an indicator of the potential presence of PRRSV in the litter, which can support decisions during PRRSV outbreak management programs. For instance, once the virus prevalence of the herd decreases, e.g., AASV Status 1B to II (2), implementing measures to prevent further spread must be considered, such as handling mortalities at the end of the day, adhering to McREBEL practices, and limiting cross-fostering protocols, especially in litters where stillborn piglets are found (31, 32).

Litter size and the number of pigs born alive are key performance indicators in pork production, as they directly impact the number of pigs weaned per sow. However, PRRSV negatively affects litter size (33). Litters with 11 or fewer liveborn piglets per sow demonstrated 12.2 times higher odds of yielding a PRRSV RNA-positive result (based on IB, TF, or BS) than larger litters, and 10.8 times higher for detecting at least one PRRSV RNA-positive liveborn piglet. For instance, in a scenario where a breeding herd is under a PRRSV elimination process with herd closure following the AASV PRRSV elimination guidelines, a target sampling focusing on small litters can be adopted to improve the monitoring program, similar to those reported for FOF and PF (16, 17).

Both herds had TF-positive results in 22 litters: five out of 26 litters in Herd A and 17 out of 104 litters in Herd B. Since TF is typically collected as an aggregated sample from multiple animals, if they had been collected as an aggregate sample in this study, i.e., all tongue tips in one bag, both herds would likely have yielded PRRSV RNA-positive results, effortlessly assessing the population’s PRRSV status. According to a previous study (34), PRRSV RNA detection in BS is comparable to serum from the jugular vein, with the advantage of being practical and less time-consuming. When PRRSV prevalence is as low as 4%, sampling 89 liveborn piglets with BS samples is needed to detect at least one PRRSV RNA-positive result. Based on BS PRRSV RNA results in this study from liveborn piglets, the liveborn piglet positivity was 3.42% (59 positives out of 1,723 sampled piglets). Thus, collecting tongue tips from multiple piglets into a single aggregated TF sample – such as from 30 to 100 individuals (6) – offers not only accurate PRRSV detection but also serve as a more time-and cost-effective alternative to individually sampling and testing 89 liveborn piglets at a prevalence of 4%. However, further studies are needed to estimate the probability of testing PRRSV-positive using RT-qPCR in an aggregated TF sample, particularly when only one tongue tip is PRRSV RNA-positive among an aggregate of negative tongue tip samples.

In this study, two PRRSV high-prevalence herds were selected to evaluate the effects of stillborn TF results and litter size comparison for PRRSV-RNA detection by RT-qPCR, aiming to maximize PRRSV-RNA positive results. This study is a proof of concept, and external validity is limited to herds sharing similar characteristics and PRRSV infection stage as the study herd. Due to internal farm management practices, most of the sampled litters were from young parities (e.g., first parity), which did not allow the assessment of the parity effect. Nevertheless, based on the disease’s ecology, stillborn piglets would still be expected in lower-prevalence herds, as virus-harboring dams continue to produce stillborn piglets, especially in the lower parity population (35).

In conclusion, under the conditions of this study, sampling litters with stillborn piglets or small litter sizes provided a higher probability of detecting PRRSV RNA-positive in the farrowing room’s population. Moreover, TF samples from stillborn piglets were a reliable indicator of the PRRSV status of their liveborn littermates, indicating that TF can be an effective risk-based approach to assess PRRSV circulation within liveborn piglets. Therefore, veterinarians and pig producers are encouraged to collect TF, targeting stillborn piglets or small litters to increase the likelihood of detecting PRRSV. This risk-based sampling strategy within the first hours post-farrowing enhances the effectiveness of PRRSV monitoring programs in breeding herds that support timely interventions, such as McREBEL and cross-fostering (31, 32). Lastly, while TFs were individually collected in this study to test the hypothesis, collecting them as an aggregated sample, similar to PF, is recommended (6), as this approach allows for a larger number of screened animals.

## Data Availability

The raw data supporting the conclusions of this article will be made available by the authors, without undue reservation.
